# A new normal in primary care: the rapid normalization of a major eHealth program in public health centers

**DOI:** 10.1186/s12913-024-11913-0

**Published:** 2024-11-15

**Authors:** Karl Maack, Nanna Gillberg, Ewa Wikström

**Affiliations:** 1 Institute of Medicine, University of Gothenburg, Gothenburg, Sweden; 2 Department of Business Administration, University of Gothenburg, Gothenburg, Sweden

**Keywords:** Implementation; eHealth; Telemedicine; Primary care

## Abstract

**Background:**

This study aimed to contribute to a better understanding of the context, mechanisms and outcomes in the implementation process of an eHealth video consultation program in primary care. The study focused on how the program is normalized in the primary care setting. The primary research question for this study is “in what ways is the implementation of video consultation normalized in primary care?”.

**Methods:**

The qualitative design and content analysis of primary data from the transcripts were based on in-depth interviews, complemented with free-text answers to open-ended survey questions and various documents. The study focuses on the large-scale implementation of the public eHealth program Närhälsan Online, which represents more than 100 health centers in Sweden’s largest region of Västra Götaland. Multiagent perspectives on how the program is normalized were drawn from expressed perceptions by professions directly linked to both strategic and functional implementation, as well as administration and clinical operation.

**Results:**

This study both confirms and enhances the field with a theoretical contribution in six ways to the reviewed previous research, as well as showing practical implications. It also provides multi-agent perspectives on the rapid normalization of the implementation program studied.

**Conclusions:**

In relation to the rapid progression of different initiatives in eHealth, this study contributes to perspectives on specific challenges as expressed by professions directly linked to both strategic and functional implementation as well as administration and clinical operation.

**Supplementary Information:**

The online version contains supplementary material available at 10.1186/s12913-024-11913-0.

## Background

### Digitalization and implementation of video consultation in primary care

Digitalization may provide opportunities for people to achieve good and equal health and welfare, as well as to develop and strengthen their own resources for increased independence and participation in society [1]. There is pressure on governments to implement new types of service provision, and with eHealth’s potential to influence cost savings in primary care, support patient access and availability to care, lessen the health burden in certain sensitive patient groups from traveling larger distances, and lessen the contagion risk at health centers. eHealth has been seen as a policy tool for managing issues connected to an aging population [1, 2]. Thus, there is growing interest from governments in how to implement eHealth and telemedicine [3].

Even if eHealth services such as telemedicine solutions have been around for a long time, especially in more analogous solutions in less densely populated countries and within transportation such as seafaring, the recent decades of rapid technological innovation have created increased opportunities for primary care to be exploited through digitalization and eHealth [1, 4–7].

A precondition for eHealth to be feasible and efficient is that they are supported by an effective infrastructure and that sound investments are needed [8]. Studies show that the emergence of remote solutions in primary care is transforming many healthcare settings and indicate that the net consumption of healthcare is increased when certain eHealth services are introduced [9]. Several studies have produced insights into the effects of implemented eHealth programs through registry-based statistics and evaluations at the macro level [10, 11].

In this study, we are particularly interested in the implementation of telemedicine, specifically video consultations that has seen an increase in primary care, especially in recent years during the global COVID-19 pandemic [8, 11]. Implementation science is inherently interdisciplinary, and to relate processes to implementation strategies, it is important to relate to both the micro individual and interpersonal, meso organizational and macro policy levels, as well as the interdependence of these different levels within the health system [12].

One challenge in implementation science is to understand the behavioral changes that occur in an implementation effort at the individual and group levels [12]. When studying the operationalization of an implementation process, it is important to understand what factors contribute to the individual and collective processes involved [13]. This includes how complex interventions are being operationalized into routines and how they are practically sustained over time. It has been suggested that more investigations on how eHealth affects roles and responsibilities, as well as risk management and effects on how to engage with professionals, are needed [14].

Previous research has reported that patients and clinicians differ in their opinions, with both experiencing positive and negative aspects from using video consultations. [15]. Clinicians consistently show a preference for face-to-face visits [15], even though there seems to be a lack of evidence when comparing clinical time within the clinical workflow. [16]. Recurring arguments for this preference include poor physical exam capabilities, reduced ability to choose correct investigations, and challenges in assessing mental health patients [17]. There seems to be a correlation between provider satisfaction if they have input into the development of the digital solution, if there is administrative support, if the technology is reliable and easy to use, and if there is adequate reimbursement for its use [18]. Resent reviews highlight the importance of sufficient guidelines and training for using the technology and that stakeholder engagement is an enabler of stakeholder trust in the technology [19].

Regarding patient satisfaction, there seems to be some consistency with video consultations being reported as correlating to the convenience of decreased travel times and costs as the main drivers for satisfaction [7, 15, 18].

One critique within implementation research is the suboptimal use of implementation frameworks throughout the phases of an applied implementation effort [20]. Rigorous and robust coding manuals are rarely described in implementation research, even though they would be helpful in understanding the analytical processes that led researchers to empirically derived insights about the implementation efforts studied. [20].

Another weakness in the discussed literature is the absence of reviews that cover the factors that promote or inhibit user engagement and participation in the successful implementation of new technologies. There is a need for more empirical studies and the need to examine the impact of eHealth services on everyday clinical practice [14].

### The study setting: Swedish primary care

Swedish primary care has the overall goal of “good health and care on equal terms for the entire population”, and is structured around the Beveridge model where healthcare is financed by income tax but provided by a mix of governmental and private actors [21]. This is a challenge under the constraint that all publicly funded healthcare must be organized so that it promotes cost-effectiveness [21]. To a large degree, the Swedish Primary care is funded and governed on a local level through income taxation in 21 counties (regions), as well as 290 municipalities, even though the overall statutory framework is decided nationally through the Ministry of Health and Social Affairs [22]. Hence, the healthcare system for primary care is decentralized to a high degree, which means that it is managed and run either by the regions or municipalities with slightly less than half being delivered through private actors.

Primary care in Sweden is regulated mainly through The Health and Social Services Act [21], with the support of The Social Services Act [23], The Act Concerning Support and Service for Persons with Certain Functional Impairments [24], The Patient Safety Act [25] and The Patient Act [26]. According to these regulations, primary care in Sweden should “be of good quality with a good hygienic standard and meet the patient's need for security, continuity and safety”, “be easily accessible”, “build on respect for the patient's self-determination and integrity”, and “promote good contacts between the patient and the healthcare staff”. With the introduction of innovative solutions all of these goals need to be considered.

Whereas most countries have been actively working to adapt their healthcare to new opportunities, the Swedish government has stated a strong vision for 2025 regarding these new opportunities:

*“In 2025, Sweden shall be the best in the world at using the opportunities offered by digitalization and eHealth to make it easier for people to achive good and equal health and welfare, and to develop and strengthen their own resource for increased independence and participation in the life of society”* [27].

Sweden has a history of being at the forefront of digitalizing healthcare [28], and Sweden has been a strong promoter of the implementation of eHealth in primary care, producing strategies in collaboration with municipalities and county councils and regions [27]. Although eHealth is continuously being implemented in Swedish primary care, it has faced criticism from medical professionals in Sweden. Issues that have been discussed include the need for physical examination, the overprescription of medicine, the underuse of diagnostic tests, and the risk of encouraging the overconsumption of healthcare [1]. Additionally, payment systems have not adapted fast enough to new ways of delivering primary care through eHealth services, which has created political tension in relation to public and private actors [1]. Another change that has been highly debated is the disruption of the traditional patient–provider power balance discussed in concepts such as “co-care” and “expert patient knowledge” [29].

Since Swedish primary care is to a large degree decentralized and consists of roughly equal parts private and public actors, there is ample of opportunity for a broad range of solutions being implemented at different health centers at the same time. The public procurements for primary care are operated at a regional level and decisions to implement new solutions can often fall on the manager of each respective health center.

This study focuses on the 104 public health centers of the largest region Västra Götaland, all operated under the mutual brand “Närhälsan”. In Sweden a lot of debate arouse when some private initiatives introduced video consultations at scale. Much of the discussion focused on the reimbursement system, which was not adapted to the new solutions. Additionally, there were concerns about meeting perceptions of what constitutes high-quality healthcare and how new initiatives might prioritize accessibility at the expense of other important factors.

Närhälsan decided to launch their own video consultation service in the form of Närhälsan online to meet the perceived demand. The decision to create a major public service was rather sudden with a full-scale implementation despite widespread skepticism and concerns about the lack of an evidence base. The implementation of this new service in this setting were chosen as study object.

### Aim of the study

This study aimed to contribute to a better understanding of the context, mechanisms and outcomes in the implementation process of an eHealth video consultation program in primary care. The study focused on how the program is normalized in the primary care setting. The primary research question for this study is “in what ways is the implementation of video consultation normalized in primary care?”.

### Normalization process theory as a translational framework

With the aim described above, this study utilizes normalization process theory (NPT) as a guiding framework in the analytical process. NPT, as described by Carl May, has been suggested and used as a framework to analyze data on eHealth implementation [13].

In NPT, actors that form implementation processes are the focus of interest, especially the individual and collective processes that occur. According to May, normalization occurs when actors seek to translate their strategic intentions into ensembles of beliefs, behaviors, artifacts, and practices that create change in the everyday practices of others [30]. These beliefs, behaviors, artifacts, and practices are not the same in each case and can be very different both over time and in different settings. This highlights the importance of the agents involved, which also relates to the NPT’s origins in the implementation of interventions in healthcare due to its stakeholder complexity.

Previous research on the implementation process has utilized the NPT to further investigate sense-making (coherence), relationships (cognitive participation), enacting work (collective action), and appraisal (reflexive monitoring), which has led to insights into organizational challenges [14, 31]. It has also been pointed out that NPT lacks consideration of the patient perspective [32], but since this study does not include patients as respondents that is not considered when applying NPT.

NPT has been further developed by Carl may to also address factors related to the context as well as the outcomes of the implementation [13]. Contexts are described as events in systems unfolding over time within and between settings in which implementation work is done and mechanisms motivate and shape the work that people do when they participate in implementation processes. An implementation process also leads to outcomes, which are the effects of implementation work in context — that make visible how things change as implementation processes proceed. With its focus on the agents involved in the implementation, the NPT centers on three questions: “What is the work that actors do to create change?”, “How does this work get done?” and “What are its effects?” [30]. This aligns well with the main research question and the aim of the study. Even though this study differ in stance when leaning to a relativistic interpretative paradigm compared to Carl Mays realist approach [13], the authors believe that this NPT configuration is providing guidance and transparency in the early coding stage, as a analytical support for the researcher in relation to implementation processes and the actions going on between agents involved. Previous research have indicated that NPT offers the researchers a framework for explaining critical implementation processes and in understanding the implementation and evaluation of interventions in recourse-constrained contexts such as primary care [32].

**Fig. 1:**
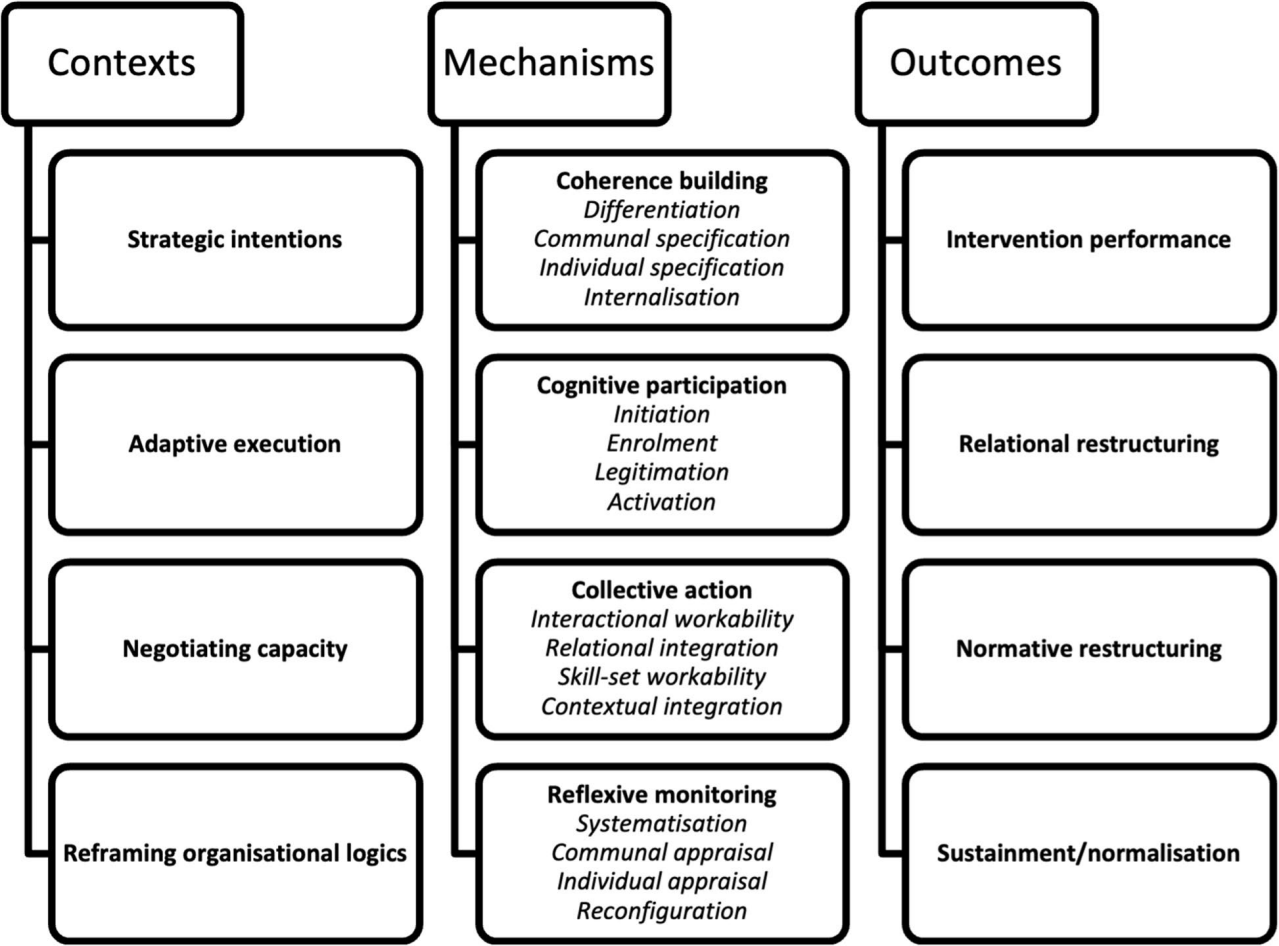
Theoretical constructs of normalization process theory. This figure outlines the theoretical constructs related to the context-mechanisms-outcome configuration (with subconstructs in parentheses)

In this study the constructs and sub-constructs of NPT serve as an overarching guide for the study design and as a focus for the interviews and specifically the subsequent coding process. In this sense, the NPT was used as a translational framework as described by Carl May, where he also defines each construct and relates it to a a coding manual [13]. An overview of all the constructs can be found in Fig. 1.

## Methods

This study answers the call of Mair et al. [14] for more empirical studies on the impact of eHealth services on everyday clinical practice. By interviewing the different agents involved in the implementation process, as well as agents utilizing the eHealth service in their clinical practice, the results contribute more insights from both individual and agent-agent collective perspectives.

### Study design

A qualitative approach has been applied as a micro-meso explanatory embedded case study design to identify, characterize, and interpret empirical deviations and regularities in the specific case of Närhälsan online. With the aim of gaining new knowledge on agents’ perspectives, the implementation process is viewed with a perception lens from different participating agents. Therefore, this study leans on a relativist ontological stance and epistemological subjective view. Our study falls within an abductive research logic within a interpretivist research paradigm [33], where we utilized NPT as a guide in the analysis of the perceived experiences from multiple agents involved in the implementation process.

Our analysis uses normalization process theory (NPT) to provide clarity and structure to the analytical process. Although Carl May developed the theory and its context-mechanism-outcomes configuration from a realist ontology, the authors believe it is suitable as a foundation for theory-driven thematic content analysis of interview transcripts, complemented by text responses to open-ended survey questions.[13].

### Object of study

The eHealth service Närhälsan Online and its various components were selected as the case for this study because of several interesting characteristics. As an eHealth service, Närhälsan Online represents a platform and service for booking and performing video consultation meetings with professions such as medical doctors, nurses and psychologists at health centers. It is available online via the Internet, as well as in a stand-alone app for mobile devices. It functions as a digital call center and operates seven days per week.

With several private initiatives challenging traditional ways of public primary care, Närhälsan online is one of the first public programs in the Swedish context. It is also a large implementation effort that covers Sweden’s largest region, Västra Götaland, and its 104 public primary health centers. The implementation is carried out by a regional team in collaboration with an externally procured private platform provider and developer. The implementation has gradually expanded over time to encompass all the public health centers in the region Västra Götaland, covering both rural and urban areas of western Sweden.

### Sample and data collection

To explore agent perspectives on the implementation of video consultation at public primary health centers, qualitative empirical data collection primarily focused on semi-structured interviews with agents involved in the implementation process. Interviews were performed via video conference and recorded for detailed transcription purposes with consent from respondents, complemented by short notes on paper. Interviews varied in length from thirty minutes to one hour and forty-five minutes.

The interview study utilized a purposeful sampling strategy with an emphasis on intensity variation and deviance to illuminate both the typical and the unusual perspectives on the case being implemented [34]. Diversity in relation to agent roles was seen as important in the selection process with the intention of supporting a breadth in data and perspectives in the analysis. The recruitment also focused on a vertical diversity with the aim to include both director level manager, middle manager, first line manager project manager, and specialists such as administrators, IT-support and coordinators, as well as representatives of the professions utilizing the services. As listed in the limitations, this study did not include patients as respondents.

The overall number of interviews (found in Table 2) was governed by the saturation of data [35], where the authors did not pursue more interviews when transcripts started to become repetitive. The number of interviews was also restricted by the availability of agents in roles that have fewer individual actors (i.e., higher director or strategic positions might only have one or two possible agent representatives employed). The recruitment was also somewhat restricted due to the strain on healthcare during the COVID-19 pandemic, which made contact difficult.

In addition to the interviews, complementary data were used from a survey conducted on the same primary care centers during the same timeframe. The survey primarily yielded brief responses to open-ended questions, while the interpretation of the transcripts was considered secondary data. The interviews and questionnaires were conducted from the late spring to the early fall of 2022. An overview of the number of respondents from specific categories of agents is presented in Table 2, found in the results section.

The full survey was part of another study, which primarily focused on attitudes of professionals, but also included open-ended questions that could provide insights on the implementation from medical doctors, nurses and psychologists employed at the health centers where Närhälsan Online was implemented. The survey covered different areas and included 42 questions, of which 6 open-ended questions were used in this specific study. A previous version of the questionnaire was developed in a prior published study focusing on the attitudes, barriers, and concerns regarding telemedicine among Swedish primary care physicians [36]. This paper utilized a new version of the questionnaire sent to physicians, nurses and psychologists with the addition of the following open-ended questions: “If you used video consultations, how do you think the consultation environment affects the meeting with the patient compared to a regular physical meeting (positive and/or negative comments)?” and “What do you think are the biggest challenges in using video calls for patient meetings in primary care?”.

Notably, the first author conducted the interviews and performed the questionnaire survey, including all the transcriptions and the main coding. This was seen as an important parameter to reach a stronger alignment between samples when translating the transcripts into codes and gathering insights in the analysis. The semi-structured interviews utilized an interview guide developed by the interviewer and tested in collaboration with the other researchers involved. Interview participants were notified that the interviewer was performing the study as part of a PhD project at the Institute of Medicine at the University of Gothenburg. The recruitment of respondents was coordinated in collaboration with the Region Västra Götalands Research and Development Office and initiated through inquiries with information about study participation, opportunities for questions and subsequent consent. A COREQ checklist for qualitative studies was used to assure quality standards [37].

### Data analysis

The interviews were recorded after obtaining consent ensure detailed transcripts, which were then imported into a qualitative analysis tool (NVivo) together with questionnaire text answers for coding and thematic analysis [38]. Overall, the coding manual for qualitative research developed by May et al. (2022) was used as a translational framework and a guiding tool to structure the process, as well as to provide stronger transparency between the raw data transcripts and the final themes [13]. With this structured theory-driven element in the coding process, the analysis focused on the perspectives of different agents toward the implementation of Närhälsan online to gain insights into the context, mechanisms, and outcome of the implementation process. An overview of the coding process and examples can be found in Table 1.

**Table 1:** Overview of the coding process. Examples of how meaningful units are translated into NPT-driven codes and then thematically analyzed in relation to different agent perspectives of the implementation process. When relating different agents’ perspectives to each other, overall thematic insights can be outlined

Meaning unit	NPT-driven coding	Agent	Theme
“I do not know what it is with healthcare workers, but when you step into the workplace, they drop all their digital knowledge…. At home they shop online and pay their bills and buy medicines, so they do everything on their computer. When they come to work, it becomes very difficult. So I think that the vocational or professional dig…, well. We have no digital maturity, and we have to work a lot on that.…”	**Skill-set workability** Code theme: A need to relate lack of digital literacy to behavioral intentions of pursuing a career in healthcare	Director of regional area (interview 5)	Relate digital literacy to behavioral intentions of pursuing career in healthcare as a potential way to attract new talent
“Some want to work in this way, more flexible and not. So you can also attract new employees by offering new roles or new types of competencies. However, it is a journey that we are just at the beginning of, I would say. Just at the beginning, and where we need to become better at defining”	**Cognitive participation/Enrollment** Code theme: A new way of attracting talent, related to new types of interests and niche intentions toward career.”	Digital strategist (interview 12)	

When meaningful units were identified they were also non-exclusively tagged in NVivo to all the constructs and subconstructs of the NPT configuration context-mechanisms-outcomes, previously outlined in Fig. 1. When the meaningful units were categorized non-exclusively in relation to NPT, all the researchers collaborated to identify code themes related to the implementation, focusing on the context, mechanisms, or outcomes of the process. From this coding process insights were identified as overarching themes, which were collectively agreed upon by the authors.

## Results

This section provides thematic results from the content analysis with a focus on findings related to the normalization process of the implemented services of Närhälsan Online. A overview of the number of respondents from different roles and categories for both the interviews and the questionnaire can be found in Table 2.

**Table 2:** Overview of respondents for both interview and questionnaire, along with clarification of the role categories to which they belong

Role	Role category	Interview	Survey	Total
Strategic directors	Middle managers	4		4
Head of digital development	High-level manager	1		1
Director regional county area	High-level manager	1		1
Head of Närhälsan Online	First line	1		1
Project manager	Project manager	1		1
Admin, IT-specialists, & coordinator	Specialist	5		5
Physician	Professional	1	40	41
Nurse	Professional	1	43	44
Psychologist	Professional	1	13	14

The overall structure of this section follows the context-mechanisms-outcome configuration of normalization process theory as presented by Carl May [13]. \* MERGEFORMAT Table 3 summarizes the constructs and themes, along with examples of their underlying meaningful units and corresponding reference sources.

**Table 3:** Overview of themes derived in the NPT-guided content coding from the meaningful units in empirical data transcripts, as well as the source of each example of meaningful unit

*Construct*	*Theme*	*Example meaningful units and sources*
*Implementation context*	*Intense communication strategy*	*High amount of continuous information meetings conducted.—Head Närhälsan online/Physician* *Word-by-mouth strategy focused on the positive clinicians.—Project manager* *Dedicated communications manager that follows the whole project and posts reports of all progress in relevant channels.—Head Närhälsan online/Physician*
	*“Try it first” method*	*Sceptics changed perspectives after trying out the services and became positive. – Project manager* *Easier to start out with the smaller health centers where it was possible to gather all the staff at once. – IT Specialist*
	*Program isolated and decoupled from overall strategic integration*	*Project managers had mandate to operate independently of regional development with a close relationship to service provider.—Strategic director Digitalization*
	*Close relationship with service provider*	*Fast adaptation of services and functions within services. – Project manager* *High knowledge exchange between service provider and healthcare. – IT-specialist*
	*Tight clear and continuous team*	*Same management through implementation.—Director regional county area* *Clear duo in the program top-management, one expert IT and one expert in medicine. – Project manager* *Clear communication on who are backing the program.—Head Närhälsan online/Physician*
	*High competence assurance*	*Only physicians with at least 2,5 years of clinical experience allowed to take part. – Project manager*
*Implementation Mechanisms* *Cognitive participation*	*Resistance*	*You often become a nurse to work close to patients and not to sit in front of a computer, we need to connect to professional development.—Strategic director Digitalization* *I think it’s the unwillingness to change, it is not the amount of work since its totally regulated in Sweden.—Director regional county area* *The responsibilities are very distributed, clinicians focus on individual patients and not on population-level challenges. Interview—Strategic director Digitalization*
	*Unclear new ways of working*	*Digitalization can be a bit scary and seen as better suited for others than medical professions.—Head of digital development* *It can become a large responsibility with the new ways of working on top of old ways.—Strategic director Digitalization* *Make the digital aids as natural as a blood pressure cuff or a stethoscope.—IT-specialist*
	*Resource allocation*	*Health centers are afraid that scheduling physicians for online will take away resources from where needed the most.—Coordinator/admin*
	*Engaged coworkers*	*When no volunteers for booking responsibility at health centers the managers just appoint someone randomly, which does not work.—Coordinator/admin*
	*Attitude change*	*Negative participants that had time to practice changed attitude and did not believe that they were negative before. – Project manager* *Clinicians who used to say this is bad has now changed, and the ones who do not want this instead say that they are too old and do not want to learn new ways of working.—Director regional county area*
*Implementation Mechanisms* *Coherence building*	*Increased administration*	*Important to clarify what work tasks are needed for new ways of working.—Strategic director Digitalization*
	*Communication differences*	*New communication channel alters how to communicate with patients, speech comes more into focus.—Nurse*
	*Transparent and open climate*	*Important to give participants opportunity to ventilate ideas, everyone can participate and have a say. -Head Närhälsan online/Physician*
	*Union and negotiation*	*Very important to invest time early in close collaborations with the union to solve possible staff allocation and negotiation issues. -Head Närhälsan online/Physician*
	*Branding and communication*	*Brand important both for recognition but also descriptive side so that patients understand what is offered.—Head Närhälsan online/Physician*
*Implementation Mechanisms Collective action*	*New roles*	*The role of “implementation responsible” were created based on the health centers need of close dialogue with expertise on the new services. – Project manager*
	*Booking routines*	*A big worry was that resources were lost if patients did not book timeslots dedicated to for example medical doctors.—Coordinator/admin* *A loss in professional respect from patients when they could book without physical visit at any time they wanted.—Psychologist*
	*Marketing of open bookings*	*You need to promote that the service exists and that there are timeslots open for booking.—Coordinator/admin*
*Implementation Mechanisms Reflective monitoring*	*Time to clinician most important*	*Patients should get to see a professional quickly in the process.—Psychologist* *According to patients, time is more important than which doctor you meet.—Head of digital development*
	*Balance different meeting types*	*Physical meetings will always be needed, and digital meetings might save more resources for physical meetings.—Nurse*
	*Quality assurance*	*High value on evaluating what is done, what works or not works and what creates a good flow for the system.—Nurse*
	*Time for reflection*	*The ones who put extra reflection and time preparing before being ready, those are the ones that are “flying” now.—Coordinator/admin*
*Implementation outcomes*	*Clear mandate and responsibility*	*Success from clear management of the implementation that stayed the same all through the implementation. -Head Närhälsan online/Physician*
	*Pandemic took away anti-perspectives*	*When pandemic hit, the resistance of total anti-personnel was suddenly quiet.—Head Närhälsan online/Physician*
	*Successful beach-head approach*	*Momentum in the implementation was built from small health centers that did not have larger administration, it became much easier to build benchmarks from these before the larger ones.—IT-specialist*
	*Close development with platform supplier*	*The high-speed implementation benefited from a very close collaboration between implementation management and the suppliers as a separate project outside of larger system developments.—IT-specialist*
	*Medical expertise & quality assurance*	*One of the strongest keys to success was the high level of medical expertise and quality assurance that was brought in to the duo-management team. – Project manager*
	*Restricted entry requirements*	*Important to have experience and we have requirements of specialist-competence and at least 2,5 years of experience for physicians. – Project manager*
	*Specialized medical guidelines*	*We have developed specialized medical guidelines for workflows at Närhälsan online.—Coordinator/admin*
	*Education*	*We now collaborate with the university and provide courses in the profession-educations on digital consultation *etc*.—Head Närhälsan online/Physician*
	*Only digital not allowed*	*It seems more stressful to only work digital, staff do not get as much back, the association for psychologists has restricted that you also need to work IRL, no-one should work only digital.—Psychologist*
	*Future outlook worries*	*Will there be a fallback to physical meetings or a continuous development of utilizing digital meetings.—Head Närhälsan online/Physician*

The following subsections was structured according to the NPT configuration to guide the reader on findings relating to content, mechanisms and outcomes of the implementation process.

### Implementation context

The implementation of Närhälsan online was strongly driven by a reactive strategic counterapproach to fast-moving initiatives in the private sector. Even if overarching societal challenges such as an aging population and related needs for cost effectiveness were somewhat addressed, the overall basis for initiation of the eHealth program was described as an attempt initiated by the county council or region to respond to private companies’ exploitation through certain telemedicine services.

On May 16, 2017, the region unanimously decided on a strategy for “restructuring of healthcare” (omställning av hälso- och sjukvård), which included “developing digital forms of care and services” as one of four key areas. Previous attempts to initiate programs for video consultations had been met with a negative response, and only one pilot implementation for youth centers had led to progress.

As a context for the implementation, the private initiatives as well as the parallel success at the youth centers led to a direct mission from the regional director to start a public implementation program for video consultations to meet the private initiatives, utilizing the project manager that managed the youth center program.

This clear top-down mission accelerated program initiation, with a service provider evaluation between May and October 2017, which led to a decision in late December of the same year, and the first online reception opened on January 1st, 2018. The decision was followed by a rather special setup, where the program became isolated as a separate project from the overall major strategic program of the region and therefore framed with a somewhat own autonomy in which the program managers could work very close to a service provider. This had a major impact on momentum, and management could operate in development without major regional integration as a hindering factor.

Another unusual choice was made where they created a duo-management team instead of only one main manager. In response to resistance met by clinicians in previous initiatives, the project manager from the pilot at the youth centers was complemented with a senior physician active in quality assurance topics as well as interested in new methods and technology. This duo-management team was recurrently referred to as the “dream team” not only in terms of drive, tenacity, and ambition but also in terms of a clear mix of competencies, where expert project management meets expert clinical practice and quality assurance.

*"I have never seen a project that has had such a real “rocket start” than Närhälsan Online's, of course it has to do with the project managers… …start the whole reception on January 1, 2018 after a pre-study within 6 months, this was a sensational [laughs at the same time] that does not happen very often!"*

*- Interview 1 (clinical project manager)*

Even though the implementation started with an intense focus on rapid diffusion, there were elements of resistance from clinicians. The major impact on implementation from the COVID-19 pandemic was that the alternatives to online consultations had just disappeared. The pandemic-induced societal lockdown led to an absence of critique against using video consultations since the only alternative was no consultation at all.

This was seen as a great opportunity to utilize, and the already strong communication strategy was further enhanced to reach out as quickly as possible. In this regard, much effort was put into mobilizing individuals who were interested in the program instead of focusing on the most resistant individuals.

*"with care meetings… …we started with the doctors first…. ....they talk to each other, so we have different types of doctor's meetings where everyone, where they meet, then talk and the ones who buy in will then spread it onward and then someone wants to try it, and so it goes by word of mouth, so that, I think it has spread with us.”*

*- Interview 5 (IT project manager)*

### Implementation mechanisms—cognitive participation

In the work of legitimizing the implementation, a need to process new perspectives from participants and healthcare management was identified. The new way of working through a digital concept and how to fit within the established structure and framework of healthcare was challenging and not obvious in relation to the complexity involved in running public healthcare services. Although the mission to implement the eHealth service was driven by an urgent need for swift action, this conceptual reframing required time for individuals to adapt their way of thinking.

Many of those involved in the implementation did not view the workload as a significant issue. The idea that workload posed a problem was dismissed, citing strict regulations in Sweden regarding allowable working hours. This highlights an interesting point when introducing this new way of working, namely, that the workload might be related to something other than the actual amount of implementation work needed.

The adaptation to a new way of working was met with some resistance. In the implementation process, there was a high degree of difference in acceptance when both managers and clinicians had a chance to practice and try out the new service. A demo or presentation was not enough, but perhaps more tacit experience was needed, as was some time to reflect on the experience. This might be due to the high amount of legislation and routines that are put on healthcare personnel, which might create a reluctance to change based on the need for reliability assurance in relation to safety and securing compliance.

Another reflection from clinicians was that in online consultations, they might be seen less as a person when not meeting patients physically, which might negatively affect the communication and respect between clinicians and patients.

During the implementation process, they needed to reconfigure the implementation at the health centers with closer collaboration with the application specialist. The issue was that the administrative secretary sometimes lacked a background in clinical challenges. When implementation responsibilities were simply assigned to them at a health center, it became difficult to gain traction when issues arose and to ensure rapid communication between needs and the application specialists. To address this, they established coordinators who could maintain close communication with the health centers. These coordinators, often former application specialists, acted as intermediaries with the expertise to quickly respond to common questions, resolve specific issues, and offer insights from previous discussions.

### Implementation mechanisms—coherence building

A challenge in healthcare arises when clinical staff, such as nurses, are unable to fulfill their essential roles due to an increasing burden of administrative tasks. This issue was highlighted as staff had to differentiate which tasks were part of their responsibilities while adapting to the new processes associated with the eHealth service. Another difference for the clinicians compared to how they normally worked was how the focus shifted in relation to some aspects of the patient-clinician meeting. How they expressed themselves verbally was very different and therefore very important to think about in relation to the new medium of communication.

One thing that had been difficult to change after recognition had started was the brand. Initially, the plan had been to use the mutual name of the public health centers, which was “Närhälsan”, and then add “online”. This was thought of as a good way for the public to recognize the public actors’ initiative within Närhälsan’s organization, as well as making it easy for staff to see the online initiative of their organization. This probably gave some easy traction in the beginning but was troublesome after activities started. Early on, the Närhälsan online more or less held an on-call reception, which did not include all the services of a meeting in the physical health center. The need to differentiate eHealth services more clearly from traditional services arose, but this was difficult to achieve once recognition had been established in the media and the public eye.

A major challenge at the beginning was determining how this new way of organizing could be collectively approved by the union.

### Implementation mechanisms—collective action

Regarding the resources of health centers, efforts are made to ensure that clinicians are utilized to the greatest extent possible. The new way of working online was therefore seen not only as not only with skepticism but also with apprehension as clinicians were unsure how many of the allocated time slots would be booked by patients. If these slots went unfilled, clinicians could have been utilized elsewhere, leading to unnecessary costs.. At the same time, it was not necessarily evident to the health centers that they needed to promote their new way of interacting since the patients otherwise would not know that there was any possible time slots offered. This way of promoting the offering toward the patients did not seem to align with the traditional operations of public health centers, and in some cases, some managers initially believed there was no demand from patients, even though many were unaware the service even existed. As a response to the identified context challenge from the lack of digital maturity and knowledge presented above, implementation management had started collaborating with higher education departments, where clinicians were trained. Here, modules were being developed, and curricula in digitalized care were taking shape.

One example where the challenges in the implementation process had led to new ways of organizing was when they added the online services within each health center. The need to be close to each health center during the early stages of implementation led to a new type of role as a “implementation responsible” that could operate more hands-on at each health center instead of just pointing to being responsible for each respective staff member.

### Implementation mechanisms—reflexive monitoring

According to clinicians, accessibility is more important for patients than the choice of what individual medical doctor they meet. This indicates that less waiting time is perceived as very important for patients, but at the same time, the clinicians indicated that the continuity of following the same patients is important. The implemented services were seen as foremost addressing the time-to-meeting aspect that patients prioritize but at the same time enabling a fast track to a clinician, even though it is not necessarily the same clinician you have met before.

Quality assurance seems to be complex, and one approach discussed was the quality defect costs that might arise. Overall, there seems to be a high need to evaluate the intervention performance and identifying situations where it supports high quality and instances where quality may falter, and then relate this to the cost effectiveness and accessibility of the services. Worth noting is the absence of comments on security or safety issues in the data from the people involved in the implementation process. When looking into the survey data, we found that the respondent professions had worries on the security of the platform and integrity of the patients.

From the implementers’ point of view, it seems to be very valuable to work closely with health centers and let them become well oriented before launching eHealth services. In relation to the implementation context previously presented, the perception is that even though many health centers could adapt quickly and start working quickly with the services, relational restructuring was worth some time to sink in. The perception was that the health clinics that took more time were more engaged in normalizing the new way of working than the ones that were quick to start.

### Implementation outcomes

During the later implementation process, politicians in the region decided in 2022 that at least 20% of all healthcare meetings should be digital. In a large region, such as the one studied, this represents many visits and caused concern among clinicians, as these decisions had been made before any in-depth evaluation had been carried out. It is likely that these services were better suited to some treatments than to others and that it may be better to focus any targets on different needs.

Based on the thematic content analysis, the respondents perceived the implementation as unusually rapid in its diffusion. At the same time, some of the respondents indicated being worried about what would become of this in the future, when the pandemic was long gone, and when skepticism may resurface.

## Discussion

To put this study’s results in the context of previous research, this study both confirms and develops the field. The implementation in this study indicated a constant interaction between implementation mechanisms, as well as the surrounding context and iterations based on implementation outcomes, as have been shown in other studies [39]. An overarching and recurrent finding in the data was the perception of the rapidity of the implementation, which can be related to the high priority by politicians. By showing the centrality of the multiagent approach, this study contributes to the reviewed previous research [14] and lifts normalization in five main ways based on the empirical findings from the different agents involved in the implementation process.

First, to support normalization in the implementation effort it is important to understand the factors that contribute to the individual and collective processes involved in an implementation process [13]. A central effort for the cognitive participation and enrolment is to perform an analysis of what types of agents need to be involved [40]. Computer skills and experience of healthcare staff has been lifted as barriers in several previous studies [41], and agents can differ in digital literacy and maturity. In order to stimulate collective action it is important to budget in relation to skill-set workability such as the lack of digital literacy and maturity within the parts of the organization affected by the implementation. Other studies have suggested strategically designing the layout of eHealth interventions, as well as focusing on accessibility [42].

Second, in strategic implementation efforts, the general and most prominent incentives for staff career choices could be regarded as central for supporting normalization through involved actors’ individual appraisal toward and engagement in the implementation process. This is also related to any assumptions put on staff perspectives on the overarching societal needs of the new intervention. Most professionals involved in video consultations tend to legitimize their work focused on individual patient health rather than population-level strategies. Identifying suitable stakeholders and recognizing their stakes and values should be considered [42].

Third, quality perspectives are central as high drivers and goals in implementation, and these perspectives contribute to normalization through strategic planning, staff allocation and enrollment by the implementation team. To facilitate cognitive participation and collective action, contextual integration is important in order to relate the new service (eHealth) to the major goals of organizations. It is also important to consider legitimization discussions when negotiating capacity and aligning skill-sets in the enrolment of the management team towards these goals. Here, the result contributes to the literature by showing the specific complexity arising from the varying needs of multiple stakeholders [7, 40]. Further studies have focused on a need for standards for eHealth systems and related concerns over patient data safety as well as professional liability [41]. Context dependent factors identified in this study includes how the duo-management team could negotiate capacity and provide clarity towards both clinical and digital perspectives, not the least in relation to legitimizing the implementation in relation to quality.

Fourth, the creation of arenas or meetings for discussion on implications with union representatives before initiating the project can facilitate coherence through differentiation and communal specification of implementation components, as well as negotiating capacity for the implementation. Scheduling and routines are highly important and complex in healthcare; therefore, it is very important to prepare implementation projects to determine how they can affect or potentially normatively restructure traditional ways of working in the surrounding context of specific implementation. There seems to be a gap in literature that focus on labor union challenges in relation to eHealth implementation. Previous studies focused on general healthcare settings and the health of staff [43] and that uncertainties exist on the impact of eHealth implementation on the work of health professionals [44]. Related general discussions focus on the privacy and data protection concerns for workers, even though it is not specifically focusing on impact of union views on eHealth implementation.

Fifth, readiness for change and the importance of tacit experience for users also facilitate normalization and should be considered when planning an implementation effort [40]. Attitudes seem dependent on reflexive monitoring and individuals’ time in trying out and reconfiguring to the new way of working. Indications in previous studies have shown that adaptability and cost, as well as expectations on more efficient workflow might be specifically important in eHealth interventions [41, 45].

There were no patients involved in this study but there were several comments from other agents that held perspectives in relation to patients. The authors believe there a need for further studies that takes the patients into account in the empirical foundation, which also has been mentioned in other studies [46].

This study’s practical contribution highlights the importance of budget time for legitimizing the project toward certain receivers, regarding the absence of a holistic understanding of organizations’ needs further down the line of organization. This is especially central when working with an implementation in a complex context, such as primary care, with heterogeneous receivers. It is important to decide whether integration parameters should be considered for a separate project or whether the intervention should be implemented with its own project management outside-line organization. Healthcare personnel are highly motivated and monitored in relation to safety, and new implementation efforts must secure and clarify safety-related issues. Since the budget for reaching out with new projects or programs in public healthcare is low, it is highly important to consider strategies for utilizing word-by-month. There is a strong culture in healthcare to help each other out, and networks are not necessarily always formally structured.

Directly connected to the expressed needs of the agents interviewed, future research initiatives should further investigate outcomes on a detailed level. It is not enough to measure patient flows in different health centers; it is also highly important to understand relations between the micro individual and meso organizational levels. What parameters are important and what relationships are needed to understand how to govern how these types of technologies, as well as how they influence new ways of working and how they affect the overall healthcare organization, in addition to macro societal perspectives. It would also be of interest to gain more in-depth knowledge on the patients’ perspectives, which were not included in this study. Another suggestion for further research from different institutional logics is to complement this type of microlevel study and register-based macrolevel outcome studies, as mentioned by other authors [12].

### Limitations of the study

With the aim of gaining new knowledge on agents’ perspectives, the generalization of results is limited since the implementation process is viewed with a perception lens from different participating agents.

The study design is based on a micro-meso explanatory embedded case study which applied a qualitative approach to identifying, characterizing, and interpreting empirical deviations and regularities in a specific case.

Since the data gathering was performed in 2022, some of the implementation data were derived from narrative experiences by actors who were involved in the much earlier stages of the implementation, which in turn can influence the accuracy of their statements as they remember it.

Another limitation is the representation of agents participating in the implementation effort. Since it is most likely that agents with a pro-implementation mindset have been recruited to participate in managing the effort, there is a probable lack of representation of agents representing anti-implementation mindsets among participating agents. Additionally, the study did not include patient involvement, which limits the patient perspective to secondary opinions from other stakeholders.

## Conclusions

This paper contributes to a deeper understanding of multiagent perspectives in the context of the rapid progression of eHealth services at public primary care health centers. In relation to the high focus and rapid progression of different initiatives in eHealth, this study can contribute to perspectives on specific challenges as expressed by professions directly linked to both strategic and functional implementation as well as administration and clinical operation.

From the findings, concerns are also raised about what would become of this rapid diffusion in the future, when the pandemic is long gone, and the skeptics might have gained more strength in the discussion.

In conclusion, the authors also believe that this way of using and presenting the use of a rigorous and robust coding manual is helpful when aiming for a transparent explanation of the analytical process that led to empirically derived insights by researchers studying implementation efforts.

## Supplementary Information

Supplementary Material 1.

## Data Availability

The datasets used and/or analyzed during the current study are available from the corresponding author upon reasonable request.

## Abbreviations

IT: Information Technology
NPT: Normalization process theory
