# Supplementary material for: A new normal in primary care: the rapid normalization of a major eHealth program in public health centers
Source: BMC Health Serv Res. 2024 Nov 15;24:1409. doi: 10.1186/s12913-024-11913-0 (PMC11566830; doi:10.1186/s12913-024-11913-0)
Supplement: Supplementary file 1 — Supplementary Material 1. [file 12913_2024_11913_MOESM1_ESM.pdf]

Manuscript: *Telemedicine and perceived barriers in Swedish primary care*

Consolidated criteria for reporting qualitative studies (COREQ): 32-item checklist

Developed from:
Tong A, Sainsbury P, Craig J. Consolidated criteria for reporting qualitative research (COREQ): a 32-item checklist for interviews and focus groups. *International Journal for Quality in Health Care*. 2007. Volume 19, Number 6: pp. 349 – 357

No. Item	Guide questions/description	Reported on Page #
**Domain 1: Research team and reflexivity**		
Personal Characteristics		
1. Inter viewer/facilitator	Which author/s conducted the interview or focus group?	Page 9 / paragraph 4 (Line)
2. Credentials	What were the researcher's credentials? E.g. PhD, MD	Page 1 / paragraph (Line)
3. Occupation	What was their occupation at the time of the study?	Page 1 / paragraph (Line)
4. Gender	Was the researcher male or female?	Not reported
5. Experience and training	What experience or training did the researcher have?	Not reported
Relationship with participants		
6. Relationship established	Was a relationship established prior to study commencement?	Not reported
7. Participant knowledge of the interviewer	What did the participants know about the researcher? e.g. personal goals, reasons for doing the research	Page 9 / paragraph 4 (Line)
8. Interviewer characteristics	What characteristics were reported about the inter viewer/facilitator? e.g. Bias, assumptions, reasons and interests in the research topic	Page 9 / paragraph 4 (Line)

**Domain 2: study design**		
Theoretical framework		
9. Methodological orientation and Theory	What methodological orientation was stated to underpin the study? e.g. grounded theory, discourse analysis, ethnography, phenomenology, content analysis	Page 7 / paragraph 3 (Line)
Participant selection		
10. Sampling	How were participants selected? e.g. purposive, convenience, consecutive, snowball	Page 8 / paragraph 6 (Line)
11. Method of approach	How were participants approached? e.g. face-to-face, telephone, mail, email	Page 8 / paragraph 6 (Line)
12. Sample size	How many participants were in the study?	Page 10 / table 1 (Line)
13. Non-participation	How many people refused to participate or dropped out? Reasons?	Page 9 /paragraph 1 **(no clear number, only clarifying reasons)**
Setting		
14. Setting of data collection	Where was the data collected? e.g. home, clinic, workplace	**Page 8 / paragraph 6**
15. Presence of non-participants	Was anyone else present besides the participants and researchers?	**Not reported (impossible to know in distance meetings)**
16. Description of sample	What are the important characteristics of the sample? e.g. demographic data, date	Page 10 / table 1 (Line)
Data collection		
17. Interview guide	Were questions, prompts, guides provided by the authors? Was it pilot tested?	Page 9 Both for questionnaire (paragraph 3) and interviews (paragraph 4)
18. Repeat interviews	Were repeat interviews carried out? If yes, how many?	**No, therefore not reported.**
19. Audio/visual recording	Did the research use audio or visual recording to collect the data?	Page 8 / paragraph 6 (Line)

20. Field notes	Were field notes made during and/or after the inter view or focus group?	Page 8 / paragraph 6 (Line)
21. Duration	What was the duration of the inter views or focus group?	Page 8 / paragraph 6 (Line)
22. Data saturation	Was data saturation discussed?	Page 9 / paragraph 1 (Line)
23. Transcripts returned	Were transcripts returned to participants for comment and/or correction?	**No, not reported**
**Domain 3: analysis and findings**		
Data analysis		
24. Number of data coders	How many data coders coded the data?	**Page 9 / paragraph 4.**
25. Description of the coding tree	Did authors provide a description of the coding tree?	Page 10 / Table 2
26. Derivation of themes	Were themes identified in advance or derived from the data?	Page 10 / paragraph 1 (Line)
27. Software	What software, if applicable, was used to manage the data?	Page 10 / paragraph 1 (Line)
28. Participant checking	Did participants provide feedback on the findings?	**N**o, therefore not reported.
Reporting		
29. Quotations presented	Were participant quotations presented to illustrate the themes/findings? Was each quotation identified? e.g. participant number	Yes, examples on Page 12
30. Data and findings consistent	Was there consistency between the data presented and the findings?	Yes, examples on Page 12
31. Clarity of major themes	Were major themes clearly presented in the findings?	Yes, provided in tables, for example page 12.
32. Clarity of minor themes	Is there a description of diverse cases or discussion of minor themes?	No, but the discussion tries to bring in author perspectives as much as possible.
